# The role of LPS and CpG in the farm effect against allergies, and beyond 

**DOI:** 10.5414/ALX02327E

**Published:** 2022-03-29

**Authors:** Vivian I.V. Gerretsen, Martijn J. Schuijs

**Affiliations:** 1 Department of Internal Medicine and Pediatrics, Ghent University,; 2 Laboratory of Immunoregulation and Mucosal Immunology, VIB Center for Inflammation Research, Ghent, Belgium, and; 3 Department of Pulmonary Medicine, Erasmus MC, Rotterdam, The Netherlands

**Keywords:** farm effect, lipopolysaccharide, bacterial DNA, microbiome, allergy, asthma

## Abstract

The prevalence of allergic disease has increased significantly over the past decades. Although allergies are inherently multifactorial and heterogenous; environmental, maternal, and early-life microbial exposures could strongly modify disease risk. The effects of environmental microbiota are illustrated by the “farm effect”, showing protection against asthma when children grow up on traditional farms. Recent studies have further revealed an important role for early-life exposure to a microbe-rich environment imposing lung and gut microbiome maturation and immune education, preventing allergic disease in childhood. Advances are made in the field of immunology and microbiome research, which identified entire microbial taxa, as well as specific microbial metabolites and bacterial products associated with reducing disease risk. Here we discuss the cross-talk between the microbiota and the pathogenesis of allergic disease, including bacterial products as lipopolysaccharide and CpG, in the farm effect.

## Introduction 

Over the last decades the prevalence of allergic disease like asthma, food allergy, and atopic dermatitis has increased dramatically, especially in areas with Western life styles and environmental exposures. Although asthma is heritable and polygenic, with many genetic variants contributing to disease risk, the interaction of these genetic factors with environmental factors such as, diet, allergens, and microbes appear important for disease pathogenesis [1]. It is increasingly well recognized that early-life exposure to environmental factors play a pivotal role in the development of asthma and other allergies. This is underlined by the fact that prevalence of childhood asthma is significantly greater in urban areas compared to rural environments, with the traditional farming environment showing the strongest protective effect, also referred to as the “farm effect” [2]. This protective effect can, at least in part, be explained by the high-level exposure to lipopolysaccharide (LPS), a cell wall component of Gram-negative bacteria, and bacterial DNA (CpG) on farms. Central to the effect of these exposures are maternal and early-life microbial and immune networks, which start during pregnancy when the mother’s microbiome shapes the immune system and microbiota of the developing fetus, and continues to do so during the first year of life, e.g., via breastfeeding. This ultimately regulates the microbiome and immune maturation, and influences the risk for developing allergic disease [2, 3]. Although our understanding of gene-microbial interactions in asthma has expanded, we still lack specific knowledge on the biological mechanisms as to how the “farm effect” and microbiota interact with host genetics and immune system to modify disease risk. In this review we will discuss the effects of environmental microbial exposure on allergic pathogenesis, focusing on LPS and CpG-derived immunotolerance. 

## The farm effect: Environmental microbiota diversity 

The hygiene hypothesis, as proposed by Strachan [4] in 1989, postulates that decreased exposure to environmental or commensal microbes, as is the case in modern-day urban lifestyles, is responsible for the increased prevalence of allergic disease in Western societies. Over time it has become clear that respiratory viral infections, like respiratory syncytial virus (RSV) and rhinovirus (RV) [5], as well as lifestyle changes and dietary habits could also affect the incidence of asthma later in life [6]. Moreover, genetic predisposition, especially the chromosome 17q21 risk alleles, also play an important part in the outcome of host interactions with its microenvironment (reviewed in [7]). Supporting the hygiene hypothesis is the observation of the “farm effect”, which is hypothesized to provide immunomodulatory stimuli sufficient to promote childhood immune maturation. As introduced above, studies have associated maternal and early-life exposure to farming environments or environments with high endotoxin loads with reduced incidence of allergic disease in children [8]. 

The best example of the farm effect is observed in the Amish and the Hutterite, two populations with similar genetic background but distinct farming patterns. Researchers have shown a sharply different asthma and allergy prevalence between the Amish children that live near animal barns and whose families use more traditional farming practices and the more industrialized farming techniques seen near the homes of Hutterite children. With Amish showing 4 – 5 times lower asthma and allergy prevalence [9]. This difference was reflected in different bacterial composition and levels of house dust endotoxins, which were found to be 7 times higher among the Amish. In addition, the proportions, phenotypes, and functions of innate and adaptive immune cells were different between both farming practices [10]. Interestingly, dust from Hutterite barns is similar in microbial content to that of Amish barn dust, and not surprisingly showed similar protection when tested in animal models [9]. A recent study in Finnish birth cohorts has revealed that in children growing up in non-farming homes, the risk of developing asthma decreased as the similarity of their home microbiota composition increased towards that of farming homes [11]. These data were replicated in German children living in rural non-farming homes, whose asthma risk was observed to be reduced when the microbiota composition was more similar to the Finnish farm homes [11]. This illustrates that it may not only be the bacterial load, but rather the microbial diversity that determines protection against asthma. 

The importance of environmental microbial exposure was further found in studies comparing Finnish and Russian children. Here, higher microbial load in the Russian drinking water was associated with allergy protection in Russia [12, 13]. Similarly, in a recent study with Croatian school children receiving drinking water from individual wells, a higher cumulative bacterial load in drinking water was associated with lower life-time prevalence of allergic disease [14]. It is important to note that the interaction between environmental microbes and allergic disease is not limited to children. In a recent large-scale house dust microbiome study, a U.S. adult farming population was sampled. Bacterial community profiles were less diverse in homes of atopic individuals, compared to controls. Additionally, specific bacterial taxa were abundantly present in house-dust of atopic patients: Cyanobacteria, Bacteroidetes, and Fusobacteria. In contrast several taxa of Firmicutes were more abundant in homes of individuals without atopy [15]. In all, these data illustrate that the bacterial composition in environmental exposure greatly affects the risk of developing atopic disease or asthma. However, other nonbacterial microbes, like fungi, may also play a role in farm effect and protect against allergic disease; however, these mechanisms are still unclear and likely involve complex microbial ecologies [16]. 

## The farm effect: Instructing human immunity and microbiome 

Farm animals provide a large microbial diversity, a relevant aspect in the farm effect. An import marker of bacterial exposure is the level of endotoxin containing LPS, an immunomodulatory molecule that binds to TLR4 and thereby induces the release of pro-inflammatory cytokines [17]. However, a dichotomy exists where LPS can either promote or prevent Th2-driven allergic responses. This dual role is illustrated in a study by Kaur et al. [18] showing that local LPS drives protection from allergic inflammation in the presence of GM-CSF, whereas a Th2-favorable effect was observed in the absence of GM-CSF. In addition to LPS, another microbial product with marked immunomodulatory properties is bacterial DNA. Bacterial DNA contains unmethylated cytosine-guanine (CpG) dinucleotide motifs, which binds to TLR9. Hereby, CpG potently activates innate immune responses and induces the production of IL-10, type I IFN, IL-12, and suppress Th2 cytokines, having the potential to reverse type 2 responses [19]. These data suggest that exposure to bacterial products, such as LPS and CpG DNA, present in the environmental microbial cocktail seen on farms may favor protection to Th2-mediated allergic disorders. Several strategies using synthetic CpG oligonucleotides (ODNs) for the treatment or prevention of allergic disease, have been studied in both animal models and human trials. Despite the positive results of preclinical studies, trials in patients evaluating the effect of CpG ODNs on allergic disease have returned mixed results [19]. 

Besides the mere release of cell wall components, environmental microbes can also colonize and change the host microbiome and influence the etiology of asthma [20]. In urban settings, the indoor microbial environment is largely composed by the human skin microbiota, resulting in people being exposed to the relative low diversity of their own microbes [21]. Interestingly, exposure to urban green space was sufficient to ensure transfer of environmental microbes, allowing the microbial composition of the skin to become more similar to soil microbiota and nasal samples to become more closely related to the air microbiota [22]. Other studies showed that farm living increased microbial diversity in mattress dust but also in microbes colonizing nose and throat [23]. These data support the idea that a combination of microbes or a diverse composition of the microbial environment offers an increased protection against atopic disease. Experimental evidence in mice has also revealed that intranasal administration of different bacterial strains (*Lactococcus lactis*, *Acinetobacter lwoffii*, or *Staphylococcus sciuri*), present in cowsheds, could protect from allergic airway inflammation in a TLR-dependent manner [24]. In-depth analysis of the Karelia study revealed that when comparing skin and nasal microbial networks, these were richer and far more diverse with high abundance of *Acinetobacter* in the Russian subjects and correlated with suppression of innate allergic immune responses [13]. The latter hints towards a role for microbiota sharing between hosts, which has also been observed with household pets that have been associated with increased bacterial composition affecting the human microbiome [25]. This close contact transfer of microbes may also explain why the “farm effect” can be passed on from mother to child, when shedding of the maternal microbiome occurs upon delivery and early childhood [26]. 

Moreover, a recent birth cohort study showed that the maturation of the gut microbiome during the first year of life may contribute to the protective farm effect [27]. This study found inverse correlations between asthma and levels of the fecal short-chain fatty acid (SCFA) butyrate, as well as with bacteria that could produce butyrate. Similarly, Gio-Batta et al. [28] found that children living on farms have higher levels of fecal SCFA acid compared to non-farm living children, which was associated with lower incidence of eczema. This study thereby touches upon the importance of the gut-lung axis and asthma protection through microbiome-produced metabolites (reviewed in [29]). The importance of the lung-gut axis was further highlighted by studies in humans associating the pro-inflammatory metabolite 12,13-dihydroxy-9Z-octadecenoic acid (12,13-diHOME), with microbial dysbiosis and increased risk for childhood atopy [30]. These observations where further strengthened when introduction of the bacterial epoxide hydrolase genes, responsible for 12,13-diHOME production into the mouse gut microbiome, was sufficient to reduce Treg cells and exacerbate allergic airway inflammation [31]. Moreover, using 16S shotgun sequencing, it was observed that the microbiome of infants who went on to develop allergic sensitization during childhood lacked genes encoding key enzymes for carbohydrate breakdown and butyrate production [32]. Finally, studies in a Finnish/Estonian birth cohort have revealed profound associations between regulatory T cell frequencies and colonization with butyrate-producing bacteria, which occurred earlier in life in Estonian children at low risk of developing allergic disease [33]. In murine studies it was further shown that dietary fermentable fiber content changed the composition of the lung and gut microbiota and dampened allergic disease. Metabolized fiber increased the levels of circulating SCFA, altering bone marrow hematopoiesis and seeded the lungs with dendritic cells (DCs) impaired in their ability to promote type-2 inflammation [29, 34]. 

These results show that it is not solely direct microbial exposure (e.g., LPS and CpG) but also the microbial company we keep that affects our disease risk and should be considered an important factor in the ongoing effort to reduce the prevalence of allergic disease. 

## The farm effect: When timing is critical 

Data from epidemiological studies provide ample evidence that early-life environmental exposure has a stronger effect on asthma pathogenesis. Human studies have suggested that in utero exposure to the farming environment may protect against asthma, hay fever, and eczema [35]. At birth, the adaptive immune system is still immature, and neonates have to rely on the innate immune system together with maternal antibodies for protection. The neonatal immune system is still skewed towards type-2 responses, as imposed in utero, necessary to condition the maternal immune system and prevent rejection of the fetus. Furthermore, at the first gasp of breath an early wave of IL-33 has been reported in mice and intubated infants [36, 37]. Enhanced type 2 immune responses before the age of 2.5 years further form a strong risk factor for persistent childhood asthma [38]. Interestingly, polymorphisms in the *IL33* gene or in the IL-33 receptor (*IL1RL1*), coding for ST2, are associated with childhood asthma and blood eosinophil count [39]. Exposure to house dust mite (HDM) during this period exacerbates the type-2 environment and accumulation of type-2 innate lymphoid cells (ILC2s), while suppressing the cytokine IL-12p35 in neonatal DCs, which could be responsible for allergic sensitization in early life [36, 40]. On the other hand, neonatal exposure to LPS abrogated the development of OVA-induced allergic airway inflammation [41]. 

It is becoming clear that time-dependent effects of endotoxin or farm exposure are strongest in at least two critical windows: 1) prenatal and 2) during early childhood. In most studies, this early childhood window refers to the first 3 weeks and first year of life, in mice and humans, respectively [42]. A landmark study profiled blood from 100 newborn babies over the first 3 months of life. This longitudinal sample set enabled investigators to follow immune maturation during the neonatal period. They reported that the important factors shaping immune maturation in early life are microbial exposures both from the infant’s microbiota as well as from the direct living environment [43]. These newly developed ideas have also been instrumental for a diametrical shift in guidelines recommending the timing of introduction of potential allergens. Indeed, children for whom allergenic foods were introduced early in life had significantly lower rates of both peanut and egg allergies at 3 years of age, compared to children in whom introduction was delayed [44]. Where food exposure at a young age prevents from food allergy, a birth cohort study in the United States showed that exposure to high levels of bacterial content in household dust throughout the first year of life was associated with protection against atopy and wheeze [45]. Along these lines, Roduit et al. [46] have reported that high levels of the bacterial metabolites’ butyrate and propionate in the feces of 1-year-old children is associated with protection against atopy. Together, these studies add to the notion that delaying exposure to potential allergens may be detrimental and increase the prevalence of allergies. This notion can be further extended to exposures such as farming environments and animal barns, unpasteurized milk, and daycare attendance, which seem more active when exposures occur in the first 2 years of life [5]. 

The protective effects of LPS exposure during childhood have been modeled in murine studies, however given the different asthma models used, the timing of the LPS treatment, and concentration of LPS used, the results are somewhat contradictory (reviewed in [47]). We have shown that in mice the protective effects of LPS exposure could last until adulthood [48, 49]. Something that was also observed in human studies, where the protective effects of early farm exposure could persist into adulthood [50]. Although it is difficult to exactly model the distinctions between early- and late-onset disease in humans, it should be noted that many patients present with their first onset symptoms of asthma or allergy in adult life. 

## Concluding remarks and future perspectives 

The protective effect of microbial exposure is not driven by a single factor but rather a complex interaction of microbiome, immunomodulatory effects of microbial components, and timing, coming together in an individual with its own genetic make-up and lifestyle. Herein, prenatal and early-life exposure have significant implications on the risk of developing disease in childhood or later in life. LPS and CpG DNA can modulate the human immune system affecting Th2-like responses; however, as a single molecule they have not been able to fully recapitulate the farm effect found in human setting. It is likely the diverse composition of bacteria and other microbes that make up the gut and lung microbiome, and the metabolites they produce that are necessary to optimally reduce disease risk. With this in mind, Kirjavainen et al. [11] distilled a protective “farm-like” cocktail of bacteria, including low *Streptococcaceae*, and high *Sphingobacetria*, *Clostridia*, and *Alphaproteobacteria*. 

Moving forward, one can question whether there is going to be a “‘standardized” microbial cocktail to be used in all individuals at risk. As the “farm effect” encompasses more than just microbial exposure, it is important to obtain a better mechanistic understanding of the role of the host genetic background in the host-microbiome cross-talk, to combat the asthma epidemic. 

## Funding 

We acknowledge the following funding sources for M.S.; Fonds Wetenschappelijk Onderzoek Vlaanderen (12Y5322N), Fund Suzanne Duchesne (managed by the King Baudouin Foundation), and Fondation ACTERIA. 

## Conflict of interest 

The authors confirm to have no conflict of interest. 
